# Vehicle Detection with Occlusion Handling, Tracking, and OC-SVM Classification: A High Performance Vision-Based System

**DOI:** 10.3390/s18020374

**Published:** 2018-01-27

**Authors:** Roxana Velazquez-Pupo, Alberto Sierra-Romero, Deni Torres-Roman, Yuriy V. Shkvarko, Jayro Santiago-Paz, David Gómez-Gutiérrez, Daniel Robles-Valdez, Fernando Hermosillo-Reynoso, Misael Romero-Delgado

**Affiliations:** 1 Center for Advanced Research and Education of the National Polytechnic Institute of Mexico, CINVESTAV Guadalajara, Zapopan C.P. 45019, Mexico; rvelazquez@gdl.cinvestav.mx (R.V.-P.); asierra@gdl.cinvestav.mx (A.S.-R.); dtorres@gdl.cinvestav.mx (D.T.-R.); shkvarko@cts-design.com (Y.V.S.); drobles@gdl.cinvestav.mx (D.R.-V.); fhermosillo@gdl.cinvestav.mx (F.H.-R.); mromero@gdl.cinvestav.mx (M.R.-D.); 2 Intel Labs, Intel Tecnología de Mexico, Zapopan C.P. 45019, Mexico; david.gomez.g@ieee.org

**Keywords:** IoT vision system, vehicle classification, One Class Support Vector Machine, vehicle detection, vehicle occlusion index, adaptive Gaussian mixture model, adaptive Kalman filter

## Abstract

This paper presents a high performance vision-based system with a single static camera for traffic surveillance, for moving vehicle detection with occlusion handling, tracking, counting, and One Class Support Vector Machine (OC-SVM) classification. In this approach, moving objects are first segmented from the background using the adaptive Gaussian Mixture Model (GMM). After that, several geometric features are extracted, such as vehicle area, height, width, centroid, and bounding box. As occlusion is present, an algorithm was implemented to reduce it. The tracking is performed with adaptive Kalman filter. Finally, the selected geometric features: estimated area, height, and width are used by different classifiers in order to sort vehicles into three classes: small, midsize, and large. Extensive experimental results in eight real traffic videos with more than 4000 ground truth vehicles have shown that the improved system can run in real time under an occlusion index of 0.312 and classify vehicles with a global *detection rate* or *recall*, *precision*, and *F-measure* of up to 98.190%, and an *F-measure* of up to 99.051% for midsize vehicles.

## 1. Introduction

The main goal of Intelligent Transportation Systems (ITS) for an Internet of Things (IoT) Smart City is to improve safety, efficiency, and coordination in transport infrastructure and vehicles by applying information and communication technologies. To this end, it is necessary to have systems capable of collecting road information and monitoring traffic.

Video cameras are a good choice for these tasks, because they are non-intrusive, easy to install, and of moderate cost. In addition, advances in analytical techniques for processing video data, together with increased computing power, may now provide added value to cameras by automatically extracting relevant traffic information, such as volume, density, and vehicle velocity.

According to the type of sensors (active or passive) and its location, different approaches for detecting and classifying vehicles has been developed, such as: on-road camera [1,2,3,4], rear and forward looking cameras onboard [5], low-altitude airborne platforms with vision [6,7], and non-camera on the road [8,9,10].

Vehicle detection can use several sensors and has a different meaning in this area, e.g., from a moving camera for driver assistance, or from static camera for traffic surveillance, as in our case. Thus, vehicle detection is the first step of a vision-based traffic monitoring process with one static camera. Several vehicle detection techniques have been successfully used on highways, such as frame differencing [11,12], background subtraction [13,14], optical flow [15], GMM [16,17], and others.

Usually, the next step in video processing is to track detected moving objects from one frame to another in an image sequence. Tracking over time typically involves matching objects in consecutive frames using features such as points, lines, or blobs [18], and from these track sequences, different object behaviors can be inferred. In [19], the authors present a real-time vision-based traffic flow monitoring system, where a flow model is used to count vehicles traveling on each lane and to produce traffic statistics. In the literature, the most widely used tracking algorithms are Kalman filter [20,21,22], adaptive Kalman filter [23,24], and particle filter [25].

After vehicle tracking and feature extraction, the final step is vehicle classification. Numerous techniques are available for automatic classification of vehicles, the most commonly used being deterministic methods [26,27], stochastic methods [20,28], artificial neural networks [29,30,31], and Support Vector Machine (SVM) [7,9,10,32].

Major contributions for vision-based traffic surveillance with static camera presented in this paper are: A very high performance vision-based system that improves the detection rate of moving vehicles through occlusion handling; the introduction of a metric Vehicle Occlusion Index (VOI) to measure and characterize vehicle occlusion; and the novel inclusion of OC-SVM with the Radial Basis Function (RBF) Kernel for the classification stage, where the input space of the classifier is 3D based on geometric features.

This paper is organized as follows: in Section 2, an overview of the related work in the area of occlusion handling and vehicle classification is presented. In Section 3, the procedures for the proposed system are described: vehicle detection with occlusion handling, vehicle tracking, and vehicle classification based on K-means, SVM, and OC-SVM. Experimental results are provided in Section 4. Discussion of the paper is presented in Section 5. Finally, the conclusions are presented in Section 6.

## 2. Related Works

Although, our work is related to *vision-based systems with static camera for traffic surveillance*, some works related to other close area “on road vision-based systems” are overviewed. In [1], Sivaranan and Trivedi present a detailed survey about the advances in road vision-based vehicle detection, tracking, and behaviour analysis, particularly as regards to sensors for vehicle detection and representative works in vision-based vehicle detection and tracking. In addition to classification, aspects such as features and occlusion should be studied. Another paper of interest, for vehicle-mounted camera, is Arrospide, Salgado and Nieto [33], where “a new descriptor based on the analysis of gradient orientations in concentric rectangles is defined”, involving “a much smaller feature space compared to traditional descriptors, which are too costly for real-time applications. A new vehicle image database is generated to train the SVM”. On other hand, Huang in [34] shows a detailed study about the background and uses the entropy for a motion detection algorithm, although it is a very good paper, the accuracy achieved was relatively low (53.43%).

Due to perspective effects, shadows, camera vibration, lighting changes, and other factors, multiple vehicles could be detected as a single vehicle, greatly affecting system performance. Therefore, occlusion handling is an important step after vehicle detection. There are several methods for reducing occlusions. For example, in [20], a line-based algorithm using a set of horizontal and vertical lines is proposed to eliminate all unwanted shadows; these lines are derived from the information of lane-dividing lines. In addition, fusion of the image frames acquired from multiple cameras is used in [35] to deal with the occlusion problem. Furthermore, an algorithm based on car windshield appearance is proposed in [21] to handle occlusions in dense traffic. In [36], occlusion is detected through convex regions, if occlusion is detected, then it is removed with a cutting region. In [37] the vehicle corner was used as feature to solve partial occlusion. In [38], feature-based tracking is used in intersections to handle the problem caused by the disruption of the features. In [39], the vehicle counting with perspective view is performed using two-appearance-based classifiers. Table 1 shows related works in the detection/counting stage. 

From this table and the literature, we conclude that:

- Only reference [20] has uses greater number of Ground Truth (*GT*) points in the detection than us, but they used only 3326 for the classification. Therefore, our work shows the greatest number of *GT* for classification.
- The *detection rate DR* or *recall* of 100% reported in [37] was achieved in a restricted scenario for only nine *GT* vehicles in 1000 frames; so, it’s not valid.
- The most of papers don´t give information about videos, that can be downloaded and tested; or they are too short, or not show an easy replication.
- Background models are addressed in the following highly cited articles [34,40,41,42], but all are based on assumptions that background pixel values show higher frequencies and less variance than any foreground pixel. Although the occlusion is not handled in these papers.
- Background-foreground algorithms transform input videos or photos, with occlusion handling or not, into an output space that is used for the classification stage.
- The output space delivered by the detection stage is the set of points or vectors modelling the moving vehicles.
- It is important to keep a low dimensional output space of the detection algorithms and/or the use of low-computational complexity features to improve the performance of these real-time systems.
- In [36] the occlusion is classified into partial and full visually, and convex regions were employed, reporting an improvement of the detection. However, a metric about the occlusion has not been presented.
- In [39] the occlusion handling algorithm is based on SVM, using 11 videos for training and another three for the detection of occlusion. Although this technique is novel, it uses images as elements of the input space for the SVM classifier. Therefore, it has a greater computational complexity than other techniques that use elements of less complexity than those images.
- All occlusion management algorithms should be tested with long-duration, high-frame-rate videos, 135-s videos and frame rates of 8 are relatively low.
- Vehicle ROI extraction based on GMM to reduce computational complexity is achieved in some works like [43].
- In our work assumptions such as (1) processing in the pixel domain, (2) tracking and decision at frame-level, (3) the use of low-computational complexity features and (4) processing of pixels in certain regions with high variability, are kept to reduce the computational complexity because these assumptions are crucial for a necessary future parallelization of these algorithms.
- Our work has the largest number of different scenarios for detection and the largest number of frames. In addition, traffic load and other metrics are given.

In the literature, there are many selected and extracted features [7,9,10,32,44,45,46] such as: wave length, mean, variance, peak, valley, acreage, acoustic signals, Histogram Oriented Gradients (HOG) features, the vehicle length, Grey-Level Co-occurrence matrix features, low level features, area, width, height, centroid, and bounding box. In the classification stage, these features are employed to classify the vehicles into several classes; the most used are small, medium, and large. Since 2006, SVM has been used for vehicle classification using other input spaces, and other different scenarios, such as static images [47], vehicles on road ramps [10], visual surveillance from low-altitude airborne platforms [7], on-road camera [32], static side-road camera [48], and laser intensity image without vehicle occlusion [46]. Also, in this work we focus on traffic surveillance with only a vision camera as sensor, the scenarios are multilane ways with a relative high traffic load, under different weather conditions and a variable occlusion index (see [49]). Table 2 shows important aspects of the related works in vehicle classification, including our results, and where *TPR* is the *True Positive Rate* or *Recall*, *TNR* is the *True Negative Rate*, *FNR* is the *False Negative Rate*.

From Table 2 and the here mentioned literature it can be seen that:

- Several systems used in addition to the video camera, other sensors, then different input spaces were created. Consequently, the use of a single static camera helps to maintain a low cost hardware system, and we have demonstrated that it is possible to have a high performance system.
- The test scenarios used in this work are richer than those presented in related papers.
- For traffic monitoring in Smart City IoT with a static camera located on the road-side, our system showed the highest performance and we calculated more performance metrics.

Motivation: For an IoT Smart City and particularly for video-based traffic surveillance, to have a very high-performance vision-based system that improves the detection rate of moving vehicles through geometric features and occlusion handling algorithms; the measurement of the occlusion by a metric here called VOI—Vehicle Occlusion Index— and the use of novel classifiers.

## 3. The Proposed System

**Figure 1:**
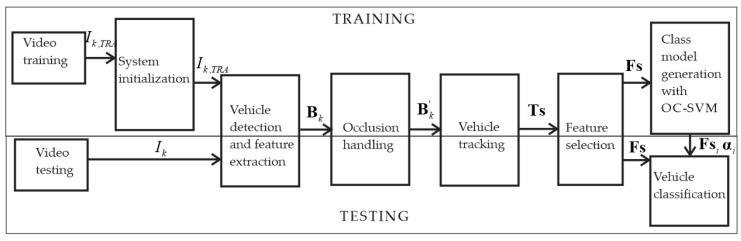
Block diagram of the proposed system.

In this paper, we present a system to detect, track, and classify vehicles from video sequences, with a higher performance than related methods in the literature. Figure 1 shows the block diagram of the system. In the training, the models for each class of vehicles are generated, for this, a training video is used. With the models, the classification is performed using OC-SVM.

### 3.1. System Initialization

**Figure 2:**
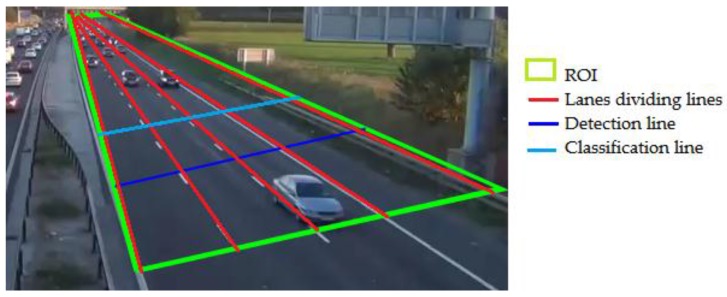
System initialization.

The tasks related with the system initialization (see Figure 2) are the following:

- Manual selection of the Region of Interest (ROI), which is the set of all pixels where moving objects or vehicles can be detected, tracked and classified. This concept helps to reduce the whole processing time.
- Manual setting of the lane-dividing lines, detection line, and classification line.

### 3.2. Vehicle Detection

It is known that different techniques can be employed for vehicle detection, e.g., pixel-domain, photo-domain. Vehicle models are built from different sets of the features, which can be geometric, based on secondary sensors, or derived by certain mathematical transformations. We will work at pixel-domain because we observed that several algorithms achieve a high performance and useful for a necessary future parallelization of the algorithms.

Although, the background modelling is not a target of this work, to have a reliable background model is a very important issue for detection of moving objects like vehicles. This problem was addressed and modelled by different authors. Stauffer and Grimson [40] developed the adaptive GMM model, while Power and Schoonees [50] revealed important practical details of this model. Mandellos, Keramitsoglou and Kiranoudis [41], and Huang [34] developed background models. Nevertheless, all of them rely on assumptions that the background pixel-values show higher frequencies and less variance than any foreground pixels. The algorithm in [41] behaves for the background as a GMM-Model, improving the foreground only working on Luv color-space that means that its computational complexity is three times that obtained in gray scale. And, as the Huang-Algorithm doesn’t show a high performance, we select the Stauffer-Grimson algorithm.

To select a background-foreground algorithm, we assume: (1) processing in the pixel domain, (2) tracking and decision at frame-level, (3) the use of some techniques to reduce the computational complexity, e.g., low-complexity features, processing of pixels in certain regions with high variability. These issues are crucial for a necessary future parallelization of detection algorithms.

Let V(τ) be a video of a duration *τ* containing *M* ground truth vehicles. It can be considered as a sequence of *K* images or frames indexed by *k* = 1, 2, …, *K*. And each frame at time *k* can be seen as a matrix, $I_{k}$ of size (m x n) where each element is a pixel value represented as $x_{k}\left(i,j\right)$, and where for the gray-space $x_{k}\left(i,j\right)\in {\mathbb{R}}_{G}^{1}$ and ${\mathbb{R}}_{G}^{1}\subset {\mathbb{R}}^{1}$ and for a 3D color-space $x_{k}\left(i,j\right)\in {\mathbb{R}}_{C}^{3}$ and ${\mathbb{R}}_{C}^{3}\subset {\mathbb{R}}^{3}$, for (1≤i≤m, 1≤j≤n). In this work, we use only the grayscale, and then the image at frame *k* is expressed as:

$$I_{k}=\{x_{k}\left(i,j\right)|x_{k}\left(i,j\right)\in {\mathbb{R}}_{G}^{1}\}$$

and the background as:

$$BG_{k}=\{x_{k}\left(i,j\right)|x_{k}\left(i,j\right)\in {\mathbb{R}}_{G}^{1}\}$$

which satisfies some mathematical background criteria.

Based on the before mentioned assumptions, the Adaptive GMM [40] was selected to segment the vehicles from the background mask. Each pixel in the image is modeled through a mixture of Z Gaussian distributions. The probability that a certain pixel has a value x at time 𝑘 can be written as:

(1) $$P\left(x_{k}\right)=\overset{Z}{\underset{z=1}{\sum }}\omega_{z,k}\cdot \eta (x_{k},\mu_{z,k},\sum_{z,k}),$$

where $\omega_{z,k}$ is an estimate of the weight of the $z^{th}$ Gaussian in the mixture at time k and η is an n-dimensional Gaussian probability density function, with a mean value μ and a covariance matrix ∑:

(2) $$\eta \left(x_{t},\mu_{k,t},\sum_{k,t}\right)=\frac{1}{{\left(2\pi \right)}^{\frac{n}{2}}{\left|\sum_{k,t}\right|}^{\frac{1}{2}}}e^{-\frac{1}{2}{\left(x_{t}-\mu_{k,t}\right)}^{\prime }\sum_{k,t}{}^{-1}\left(x_{t}-\mu_{k,t}\right)}.$$

Each pixel value, $x_{k}\left(i,j\right)$ at position (i,j) and frame *k*, that does not match the background, $BG_{k}$, is used to construct the foreground $B_{k}$, also:

$$B_{k}=\{x_{k}\left(i,j\right)|\;\mathrm{Difference}\;\left|I_{k}-\;BG_{k}\right|\;\mathrm{is}\;\mathrm{significant}\}$$

After that, a connected components analysis is performed to group those pixels that model possible vehicles embedded in the input video, and these groups are called blobs in the literature. If a frame *k* or image contains *L* groups of possible vehicles or blobs, $\mathrm{blo}b_{l}^{k}$, then:

$$\mathrm{blo}b_{l}^{k}=\left\{x_{k}\left(i,j\right)|\mathrm{pixel}\;\left(i,j\right)\;\mathrm{is}\;\mathrm{connected}\;\mathrm{to}\;\mathrm{pixel}\;\left(r,s\right),\;\mathrm{and}\;\mathrm{blo}b_{l}^{k}\subset B_{k}\right\}\;for\;l=1,\ldots ,L$$

Note that, variable *l* is used to index a possible vehicle and index *k* for its temporal behavior or frame. Then, for the video V(τ), *l* = {1, 2, …, *N*}, and where *N = M* for the ideal case. Any blob is denoted by blob, specific blob indexed by *l* is denoted as ${\mathrm{blob}}^{l}$, and temporal instances as ${\mathrm{blob}}_{k}^{l}$.

### 3.3. Feature Extraction

**Figure 3:**
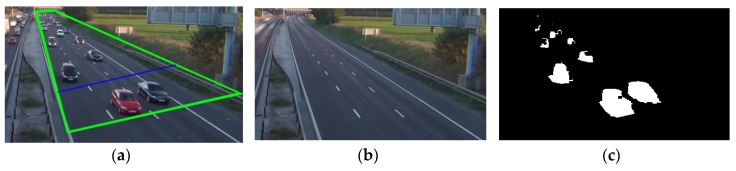
Vehicle detection: (**a**) actual image, green lines indicate the ROI, and blue line the detection line; (**b**) background and (**c**) foreground mask.

In our case, the blobs are extracted from the foreground mask, and binary morphological operations (erosion and dilation) are performed to reduce noise and enhance the geometry and shape of the objects. Next, blob analysis is used to extract geometric features such as area (the sum of the connected pixels or spatial occupancy), height, width, and centroid of the bounding box, see Figure 3. Finally, if we select *d* features as explained in Section 3.6, each blob is mapped to a new point or vector $x\in {\mathbb{R}}_{F,}^{d}$ where ${\mathbb{R}}_{F}^{d}\subset {\mathbb{R}}^{d}$ is a *new space before occlusion-handling* where the vehicle models live. 

It is important to observe the following notation. Any moving vehicle is referred as $x\in {\mathbb{R}}_{F,}^{d}$ and its temporal instances at time or frame *k* by $x_{k}\in {\mathbb{R}}_{F,}^{d}$ specific vehicle indexed by *l* is denoted as $x^{l}\in {\mathbb{R}}_{F,}^{d}$ and its temporal instances by $x_{k}^{l}\in {\mathbb{R}}_{F}^{d}$.

### 3.4. Occlusion Handling

**Figure 4:**
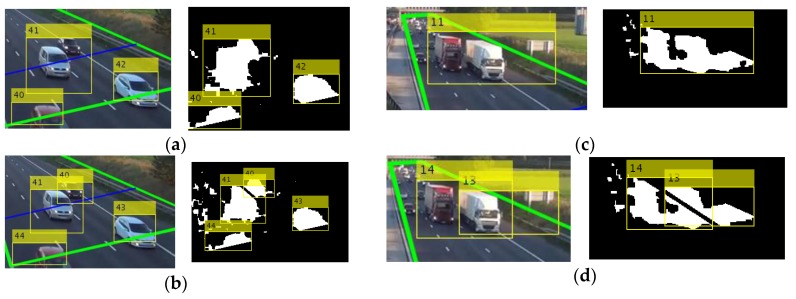
Occlusion handling when cases 1 and 2 are fulfilled, green lines indicate the ROI, and blue line the detection line. Actual image and foreground mask, (**a**,**c**) before applying the algorithm and (**b**,**d**) after applying the algorithm.

Due to camera position and height, occlusion occurs, and several errors are generated during the detection stage. The major task of any occlusion-handling algorithm in these scenarios is to minimize effects of the occlusion caused by large vehicles due to the high variance of their feature values. Therefore, we propose a simple algorithm to reduce these occlusion effects. This algorithm is based on the following assumptions:

1. The width of a vehicle cannot be greater than the width of one lane, except when it is a large vehicle that is completely inside the ROI (due to perspective effects), i.e.,:
  (3) $$\mathrm{if}\;\left(\frac{w_{b}}{w_{lane}}>Th_{1}\right)\;\mathrm{and}\;\left(\bar{a}<Th_{m}\right)\to \mathrm{Occlusion}$$
2. The width of a vehicle that is before the detection line cannot be greater than the width of two lanes, even if it is a large vehicle, i.e.,:
  (4) $$\mathrm{if}\;\left(\frac{w_{b}}{w_{lane}}>Th_{2}\right)\;\mathrm{and}\;\left(\mathrm{blob}\;\mathrm{is}\;\mathrm{before}\;D\right)\to \mathrm{Occlusion}$$
  where $w_{b}$ is the vehicle width (bounding box width), $w_{lane}$ is the lane width, $\bar{a}$ is the normalized area, D is the detection line, and $Th_{1},\;Th_{2},$ and $\;Th_{m}\;$ are the thresholds with values 1.22, 2.27, and 0.12, respectively. The values of thresholds were selected using a training video with occluded vehicles; the values that increase the detection rate were selected. If at least one case is fulfilled (Figure 4a,c), then we use the lane-dividing lines to separate vehicles traveling side by side, which are detected as a single object.

#### 3.4.1. Algorithm for Occlusion Handling Based on Lane Division

**Inputs**: D, $B_{k},$ $\left\{c_{m,k};m=1,\ldots ,M\right\}$, $\left\{a_{m,k};m=1,\ldots ,M\right\}$, $\left\{b_{m,k};m=1,\ldots ,M\right\}$, and $\left\{L_{j};j=1,\ldots ,\;J\right\}$

**Outputs**: $B_{k}^{\prime }$, $\left\{c_{m,k};m=1,\ldots ,M\},\{a_{m,k};m=1,\ldots ,M\right\}$, and $\left\{b_{m,k};m=1,\ldots ,M\right\}$,

where D is the detection line, $B_{k}$ is the foreground mask in frame $k,\;L_{j}$ is the $j^{th}$ lane-dividing line, $c_{m,k},\;a_{m,k}$ and $b_{m,k}$ are the central point, area, and bounding box of vehicle m in frame k, and $B_{k}^{\prime }$, is the updated foreground mask.

**Figure 5:**
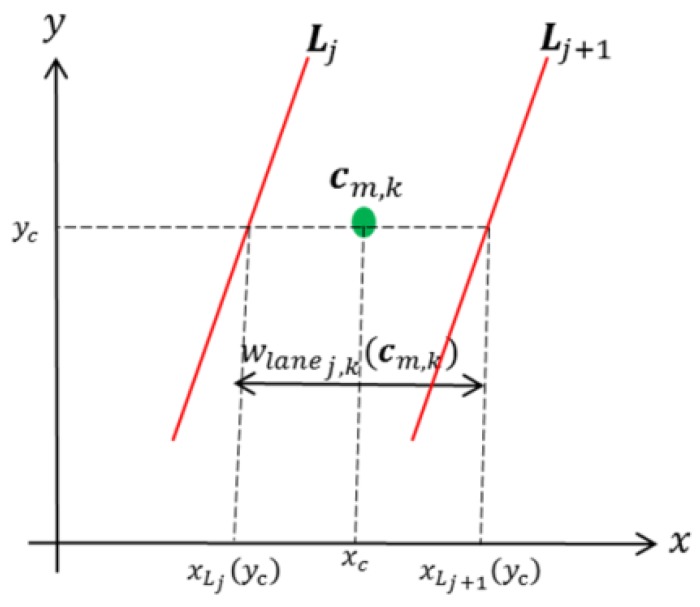
Estimation of the lane width.

For each blob $\mathrm{blo}b_{l}^{k}$ of $B_{k}$:

Step 1: Find $L_{j}$ and $L_{j+1}$ for $c_{m,k}$ (Figure 5). 

Step 2: Estimate the lane width at point $c_{m,k}$, as follows:

(5) $$w_{lane}{}_{j,k}\left(c_{m,k}\right)=\left|x_{L}{}_{j}\left(y_{c}\right)-x_{L}{}_{j+1}\left(y_{c}\right)\right|,$$

where $x_{L}{}_{j}\left(y_{c}\right)$ is the abscissa of the point on the $j^{th}$ lane-dividing line with $y_{c}$ as the ordinate (Figure 5).

Step 3: Compute the normalized area as follows:

(6) a¯m,k =am,kw2lanej,k(cm,k)

Step 4: Check if there is occlusion using Equations (3) and (4). If at least one case is fulfilled, then draw:

(7) $$\{\begin{array}{ll} L_{j+1} & \mathrm{if}\;d\left(c_{m,k},L_{j}\right)>d\left(c_{m,k},L_{j+1}\right)\\ L_{j} & \mathrm{otherwise} \end{array}$$

where $d\left(c_{m,k},L_{j}\right)$ and $d\left(c_{m,k},L_{j+1}\right)$ can be defined as follows:

(8) $$d\left(c_{m,k},L_{j}\right)=\left|x_{L}{}_{j}\left(y_{c}\right)-x_{c}\right|,$$

(9) $$d\left(c_{m,k},L_{j+1}\right)=\left|x_{L}{}_{j+1}\left(y_{c}\right)-x_{c}\right|.$$

Step 5: If all blobs have been analyzed and at least one lane-dividing line drawn, then extract the features, update the space $B_{k}^{\prime }$, and end the algorithm. Otherwise, go to step 1.

The algorithm for occlusion handling considers a static camera, and previous initialization of system, i.e., the lane-dividing lines must be defined. If the camera changes its position, it will be consider as another scenario, then the initialization of system is required. The vehicles are detected in an area of approximately 5380 ft^2^, once an object is detected, the algorithm for handling occlusions begins to work.

Challenge: The challenge of any occlusion-handling algorithm in these scenarios is to minimize the effects of occlusion caused by large vehicles due to the high variance of their feature values, delivering a uniform space, which will be the input space for the classification stage.

At this point, we will have the new vehicle space $B_{k}^{\prime }$ expressed as:

$$B_{k}^{\prime }=\{x_{r}^{s}|x_{r}^{s}\in {\mathbb{R}}_{F}^{d},\;\mathrm{and}\;x_{r}^{s}=x_{k}^{l}\;\mathrm{when}\;\mathrm{there}\;\mathrm{is}\;\mathrm{not}\;\mathrm{occlusion}\}$$

where index *s* = {1, 2, …, *S*} and *S* is the number of vehicles after occlusion, i.e., *S* *≥ N*.

#### 3.4.2. Vehicle Occlusion Index

Occlusion is an open issue in this area. Some authors classify it into total and partial and some measurements with the area are given. For vehicle traffic surveillance, under the assumption that the detection algorithm perform well, is important to know how frequent the occlusion is and how well the occlusion algorithm performs its function. As occlusion occurs in short time intervals, the measurements should be realized in the same intervals. For these purposes, we introduce here a Vehicle Occlusion Index (VOI).

The VOI-Index is defined as the ratio of the number of new vehicles detected using the occlusion algorithm and the total number of new vehicles detected during a time interval:

(10) $$VOI_{\tau }=\frac{\mathrm{number}\;\mathrm{of}\;\mathrm{new}\;\mathrm{detected}\;\mathrm{vehicles}\;\mathrm{by}\;\mathrm{the}\;\mathrm{occlusion}\;\mathrm{algorithm}}{\mathrm{total}\;\mathrm{number}\;\mathrm{of}\;\mathrm{new}\;\mathrm{vehicles}\;\mathrm{detected}},$$

where τ is the interval of time. A $VOI_{\tau }=0$ indicates that no new vehicles were detected by the algorithm or that the occlusion was not present in the time interval, while a $VOI_{\tau }=1$ indicates that the new vehicles detected by the algorithm were tracked and counted too. The VOI versus time is a measure of the frequency with which the occlusion is present. In Table 3 the average VOI-Index for the studied videos is given, while in Section 5 results of the occlusion handling algorithm and VOI-Index are discussed. 

Occlusion handling algorithms and occlusion metrics should be studied taking into account: techniques or methods used e.g., convex regions, SVM classifiers, and geometric feature space, computational complexity, classic performance metrics. In addition, they should be tested with long-duration videos and high frame rates, and should be compared with each other.

### 3.5. Vehicle Tracking

As the Kalman filter (KF) is an efficient and well known recursive filter that estimates the internal state of a linear dynamic system from a series of Gaussian noisy measurements. In mathematical terms, a linear discrete-time dynamical system embodies the following pair of equations [51]:

(1) Process equation
  (11) $$x_{k}=Fx_{k-1}+\omega_{k-1},$$
  where x is the state vector, F is the transition matrix, and ω is the process noise; the subscript k denotes discrete time instant. The process noise is assumed to be additive, white, and Gaussian, with zero mean and the covariance matrix defined by:
  (12) $$E\left[\omega_{n}\omega_{k}^{\prime }\right]=\{\begin{array}{c} Q_{k}\;f\;\mathrm{or}\;n=k\\ 0\;f\;\mathrm{or}\;n\neq k \end{array},$$
  where the superscript ${}^{\prime }$ denotes matrix transposition.
(2) Measurement equation
  (13) $$z_{k}=Hx_{k}+v_{k},$$
  where z is the measurement vector, H is the measurement matrix, and v is the measurement noise, which is assumed to be additive, white, and Gaussian, with zero mean and the covariance matrix defined by:
  (14) $$E\left[v_{n}v_{k}^{\prime }\right]=\{\begin{array}{c} R_{k}\;f\;\mathrm{or}\;n=k\\ 0\;f\;\mathrm{or}\;n\neq k \end{array}$$

Since the time of the frame interval is very short, it is assumed that the moving object is in constant velocity within a frame interval. The state in frame k can be represented by the vector:

(15) $$x_{k}={\left[x_{c,k},\;v_{x,k},\;y_{c,k},\;v_{y,k}\right]}^{\prime },$$

where $x_{c,k}$, $y_{c,k}$ are the centroid coordinates and $v_{x,k},\;v_{y,k}$ are the velocity components. The measurement vector of the system can be represented as:

(16) $$z_{k}={\left[x_{c,k},\;y_{c,k}\right]}^{\prime }.$$

For the whole video and frame by frame the blobs $\mathrm{blo}b_{k}^{l}$ represented as vector $x_{k}^{l}\in B_{k}^{\prime }$ are tracked by the corresponding Kalman filters, resulting vehicle tracking sequences $\mathrm{Ts}\left(x\right)=\left\{x_{1},\;x_{2},\;\ldots ,\;x_{k}\right\}$ as output space, where x represent any moving vehicle and $x_{i}$ are its instances.

### 3.6. Feature Selection and Environment for Classification

The detection stage delivers the whole space of tracked objects, i.e., detected vehicles or moving objects, to the classification stage. Also, all object tracking sequences, ***Ts****(**x**)*, belong to the input space of the classification stage. As each sequence ***Ts****(**x**)* includes geometric and cinematic features and their temporal behaviors, it is necessary to decide where and/or when the instances are taken for classification. Also, for each moving vehicle ***x*** corresponds a temporal sequence $\mathrm{Ts}\left(x\right)=\left\{x_{1},\;x_{2},\ldots ,\;x_{c},\ldots ,\;x_{k}\right\}$ where $x_{c}$ should be a well-defined instance of its class.

**Figure 6:**
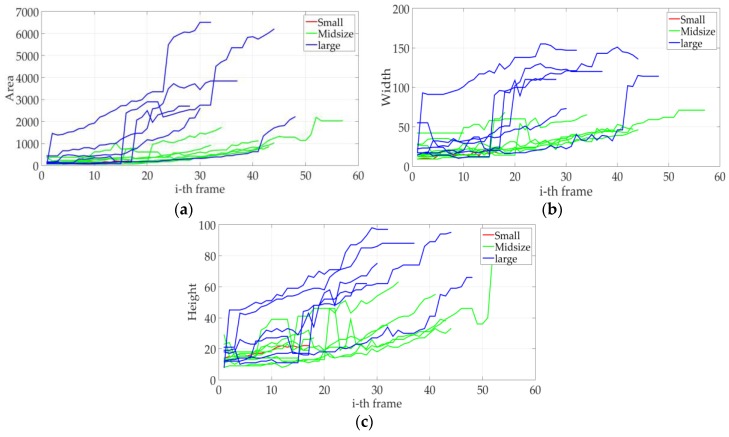
Behavior of the selected geometric features of the detected vehicles, (**a**) area of the detected objects; (**b**) width of the detected objects, and (**c**) height of the detected objects.

As these moving objects or vehicles are detected in different points of the ROI, the behaviors of the features are highly variable, and the most significant geometric feature—the area—is not sufficient for a good classification (see later Section 4.3). Studying other geometric features, such as the width and height of the bounding box, we observed that these showed a lower variance than the area (spatial occupancy). Particularly, these three features presented a very high variance for large vehicles, but a relatively low variance for midsize and small vehicles, see Figure 6.

**Figure 7:**
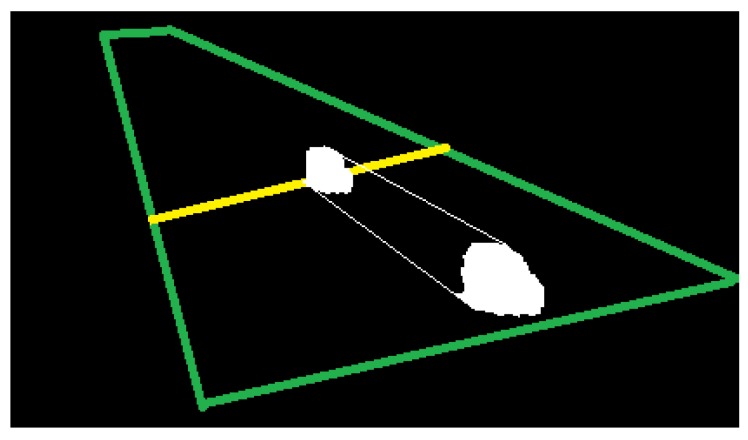
Projection of the vehicles into a classification line (yellow), green lines indicates the ROI.

As a class is a subspace of the input space, and inside of each class there are several points, and each point has several instances, is necessary to reduce these intra-class differences. Therefore, we propose for classification:

1. Instead of 1D geometric feature space, the use of a 3-D geometric feature space, ${\mathbb{R}}^{3}\subset {\mathbb{R}}^{d}$. Then, for the detected vehicles or blobs are used the input points $x\in {\mathbb{R}}^{3},\;x=\left(\mathrm{Area},\mathrm{Width},\;\mathrm{Width}/\mathrm{Height}\right)$.
2. Classification is performed in a specific line of the ROI, called here classification line, *to reduce intra-class differences* of the space of tracking sequences ***Ts****(**x**)* (see Figure 7).
3. Reduction in the variation of the feature values of any input point by using the *average of feature values* of the last three instances—detected at k-th frame after the classification line—and projecting them to the classification line, i.e., Proj(***x***).

Challenge: The challenge is to find and select significant and/or invariant features for a very high detection rate and precision under different weather conditions and for several scenarios.

### 3.7. Vehicle Classification

Classification is carried out here based on input space and classifiers:

1. 1D feature input space and thresholds.
2. 3D feature input space and K-means.
3. 3D feature input space and SVM.
4. 3D feature input space and OC-SVM.

For case 1, once the estimated area has been computed, the vehicles are classified. The decision rule for classification is defined as:

$$\{\begin{array}{cc} \text{small vehicle} & \mathrm{if}\;{\hat{a}}_{n}\leq Th_{s},\\ \text{midsize vehicle} & \mathrm{if}\;Th_{s}\leq \;{\hat{a}}_{n}\;\leq Th_{m},\\ \text{large vehicle} & \mathrm{otherwise}\;, \end{array}$$

where $Th_{s}$ and $Th_{m}$ are the thresholds for every class with values of 0.12 and 1.2, respectively. 

For cases 2, 3, and 4, the vehicles are represented by vectors $x\in {\mathbb{R}}^{3}$, which will be classified through K-means, SVM, and OC-SVM. In the classification employing the OC-SVM algorithm, a model for each class was defined. OC-SVM allows considering different behaviors of the detected blobs belonging to the same class.

OC-SVM [52,53,54,55] maps input data $x_{1},\;\ldots ,\;x_{N}\in A$ into a high dimensional space F (via Kernel *k*(*x*,*y*)) and finds the maximal margin hyperplane that best separates the training data from the origin. To do this, the following quadratic program must be solved [52]:

(17) $$\underset{w\in F,b\in \mathbb{R},\;\xi \in {\mathbb{R}}^{N}}{\min}\frac{1}{2}{\left\|w\right\|}^{2}+\frac{1}{\upsilon N}\overset{N}{\underset{i}{\sum }}\xi_{i}-b,$$

Subject to $\left(w\varphi \left(x_{i}\right)\right)\geq b-\xi_{i};\;\xi_{i}\geq 0,\;\upsilon \in \left(0,1\right],$ where w is the normal vector, φ is a map function A→F, b is the bias, $\xi_{i}$ are nonzero slack variables, υ is the outlier parameter control, and k(x,y)=〈φ(x),φ(y)〉. The equation is solved through a kernel function and Lagrangian multipliers $\propto_{i}$, and the solution returns a decision function of:

(18) $$f\left(x\right)=sgn\left(\overset{N}{\underset{i}{\sum }}\alpha_{i}k\left(x_{i},x\right)-b\right)$$

where $w=\sum_{i}\alpha_{i}\varphi \left(x_{i}\right)$ and $\sum_{i}\alpha_{i}=1.$ The kernel function used in this paper is the RBF, $k\left(x,y\right)=e^{-\eta \|x-y\|}.$

Challenge: The challenge in the classification is to find mathematical classifiers of the hypothesis set that allow mapping every point of the input space to the corresponding classes of the output space with minimal error.

## 4. Experimental Results

### 4.1. Video Processing: Test Environment

In this work, the performance of the proposed system was tested on real traffic videos: three videos, V1, V2, and V3, recorded in Guadalajara, Mexico; two videos (V4, V5) obtained from the GRAM Road-Traffic Monitoring (GRAM-RTM) dataset [56,57] (the video named V4 corresponds to video M-30, and the video named V5 is video M-30-HD); and video (V6, V7, and V8) recorded in Britain’s M6 motorway (see [58]). 

The resolution of all videos was reduced to 420 × 240 pixels at 25 frames per second and downsampling was performed to decrease the computation time. The camera’s field of view was directly ahead of the vehicles. Videos V1, V2, and V3 were recorded with a cell phone at a height of 19.5 ft on the road. This video contains double trailer traffic, which is not present in the other videos. In addition, there is quite a bit of vibration. All image frames were visually inspected to provide the ground truth (*GT*) dataset for evaluation purposes.

**Figure 8:**
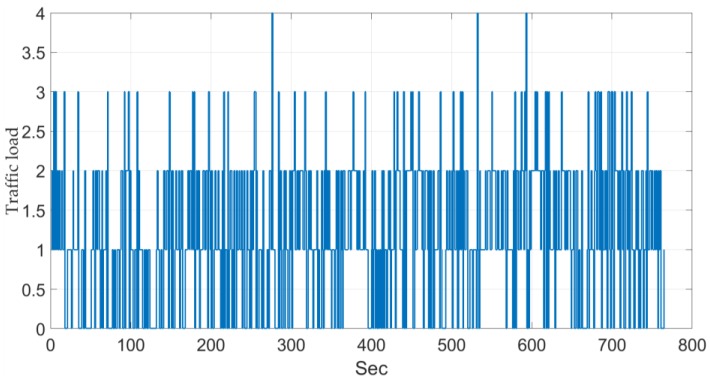
Traffic load (vehicles per second).

Table 3 shows the number of frames in each video, the traffic load, and the place and weather conditions. In addition, more than 61 min of video, 4111 ground truth vehicles, three places in different countries and weather conditions, a traffic load of up to 1.32 vehicles/s with traffic load peaks from 2 to 4 vehicles/s (see Figure 8), and a vehicle occlusion index—VOI—from 0.00 to 0.312.

The system was implemented in MATLAB and tested on an Intel Core i7 PC, with a 3.40 GHz CPU and 16 GB RAM. The metrics used to characterize the system performance in different stages are the same, i.e.,:

(19) $$Detection\;rate\;or\;Recall\;=\frac{TP}{TP+FN}$$

(20) $$Precision=\frac{TP}{TP+FP}$$

(21) $$F\;measure=2\times \frac{Recall\times Precision}{Recall+Precision}$$

where *TP*, *FP* and *FN* have different interpretations depending on the stage where they are used. In the detection stage:

- *GT* in the video is the ground truth or input space,
- *TP* is the number of vehicles successfully detected,
- *FP* is the number of false vehicles detected as vehicles,
- *FN* is the number of vehicles not detected,
- *GT’* is the output space or the set of all points detected as moving vehicle, then *GT’* is greater than *GT*.

In the classification stage, for the classes S small, M midsize and L large vehicles:

- $GT^{\prime }$ is now the new input space for classification,
- TP(class i) is the number of vehicles classified into the correct **class** i,
- FP(class i) is the number of vehicles classified into class i that belong to another class j, j≠i,
- FN(class i) is the number of vehicles of class *i* classified into another **class** j, j≠i.

For M classes

(22) $$GT^{\prime }\left(\mathrm{class}\;i\right)=TP\left(\mathrm{class}\;i\right)+FN\left(\mathrm{class}\;i\right)$$

Any point x∈FN(class i) will be classified into another ***class*** j, j≠i; then this point will be seen as FP(class j), and consequently:

(23) $$FN\left(\mathrm{class}\;i\right)=\overset{M}{\underset{\begin{array}{c} j=1\\ j\neq i \end{array}}{\sum }}FP_{i}\left(\mathrm{class}\;j\right),$$

where $FP_{i}\left(\mathrm{class}\;j\right)$ are the elements of class *i* classified as belonging to ***class j***, j≠i:

(24) $$\overset{M}{\underset{i=1}{\sum }}FN\left(\mathrm{class}\;i\right)=\overset{M}{\underset{i=1}{\sum }}\overset{M}{\underset{\begin{array}{c} j=1\\ j\neq i \end{array}}{\sum }}FP_{i}\left(\mathrm{class}\;j\right)$$

Consequently, for each ***class i***, we will have their associated metrics e.g., *DR(**class i**), Precision (**class i**)* and *F-measure (**class i**)*, which have generally different numerical values from one to another class, see Table 4, class S, M or L of any video:

(25) DR(class i)≠Precision(class i)≠F−measure(class i).

But, for the classifier with ***all classes*** we have:

(26) $$TP\left(\mathrm{all}\;\mathrm{classes}\right)=\overset{M}{\underset{i=1}{\sum }}TP\left(\mathrm{class}\;i\right)$$

(27) $$FN\left(\mathrm{all}\;\mathrm{classes}\right)=\overset{M}{\underset{i=1}{\sum }}FN\left(\mathrm{class}\;i\right)$$

(28) $$FP\left(\mathrm{all}\;\mathrm{classes}\right)=\overset{M}{\underset{i=1}{\sum }}FP\left(\mathrm{class}\;i\right)$$

and from Equations (23) and (24):

(29) FN(all classes)=FP(all classes),

Then, the following metrics, although with different physical meanings, are numerically equal each other, i.e., (Equations (19), (20) and (21), and see Table 5, for all classes of any video:

(30) DR(all classes)=Precision (all classes)=F−measure (all classes)

The most significant metrics are *detection rate* or *recall* for the detection stage and *F-measure* for the classification stage, because it works on the complete input space for these scenarios, i.e., the space including *TP*, *FP* and *FN*—see Equations (19), (20) and (21).

### 4.2. Vehicle Detection Results

Table 4 shows the experimental results of the detection stage using the occlusion algorithm. Experimental results show that the detection stage without the occlusion-handling algorithm has a *detection rate* of 83.793% (see Table A1), while that using the occlusion-handling algorithm in the detection stage improves the *detection rate* by 11.423%, and the number of vehicles detected increased to 95.216%. During the detection stage of these videos, a very strong correlation was found between *F-measure* and the measured VOI index. 

*FP* are produced by various conditions: camera locations with high vibration, camera angle, certain morphological operations embedded in the detection algorithm and because the occlusion algorithm divides large blobs into two or smaller ones, and some of them are not vehicles, i.e., *FP*. Particularly, videos V1, V2, and V3 were recorded in Mexico, where very large vehicles can transit, and the locations of the cameras showed a high vibration. The V4 and V5 videos were recorded in Madrid, Spain, showing a VOI index equal to 0 and the lowest *FP* numbers. While V6, V7, and V8 with a VOI index close to 0.2 showed results considered normal. These results show that it is necessary to improve the implemented occlusion handling algorithm, using other methods such as the convexity of the blobs and techniques such as K-means and SVM.

### 4.3. Vehicle Classification Results

**Figure 9:**
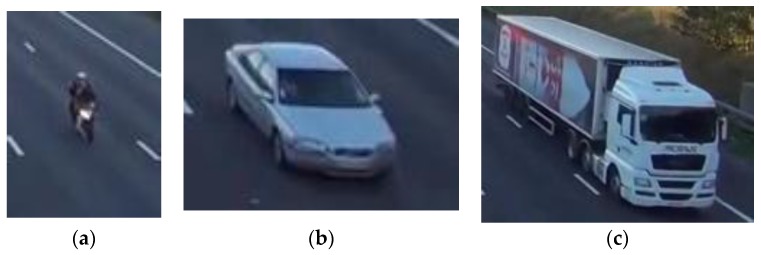
Vehicle examples for every class: (**a**) small; (**b**) midsize and (**c**) large.

The LIBSVM library [59] was used to implement the OC-SVM—and SVM—classification with a RBF Kernel. Additionally, for comparison purposes, K-means algorithm was implemented. Figure 9 shows one example for every vehicle class. 

Table 5 shows the experimental results of the classification stage (with occlusion handling in the detection stage) using OC-SVM and the three selected features (*area*, *width*, *relHW*), where S, M, and L denote small, midsize, and large vehicles, respectively.

Table 6 shows the experimental results of videos V6, V7, and V8 in the classification stage (with occlusion handling in the detection stage) using the thresholds, K-means, SVM and OC-SVM and the three selected features (*area*, *width*, *relHW*), where S, M, and L are small, midsize and large vehicles, respectively.

Experimental results show that the performance of the classifiers increases when using three geometric features. In addition, SVM and OC-SVM classifiers have better performance than K-means. By using a single geometric feature, e.g., *area*, the *recall* and particularly the *F-measure* were 77.322%. However, using 3D feature input space and OC-SVM, the *F-measure* achieved a value of 98.190%.

## 5. Discussion

### 5.1. Test Environment 

Eight videos with 4111 manually labelled ground truth vehicles and a duration of more than 61 min, three places in different countries and under different weather conditions, a mean traffic load of up to 1.32 vehicles/s with traffic load peaks from 2 to 4 vehicles/s (see Figure 8), and a vehicle occlusion index of up to 0.312. The system performs well and in real time under all these scenarios. 

### 5.2. Occlusion Handling Algorithm and VOI-Index 

As multiple vehicles will be detected as one due to perspective effects or shadows, an algorithm to reduce this occlusion was implemented. This algorithm allows improving the *detection rate* from 83.793% to 95.216% (see details in Table A1). *FP* are produced by various conditions: camera locations with high vibration, camera angle, certain morphological operations embedded in the detection algorithm and because the occlusion algorithm divides large blobs into two or smaller ones, and some of them are not vehicles, see Section 4.2, for details about videos V1–V7. From Table 3 and Table 4 we can conclude that a VOI-Index = 0 doesn’t mean that the number of *FN* is equal to 0, but indicates us that the algorithm for detection of moving vehicles should be improved. 

### 5.3. Clustering Analysis 

Clustering analysis, e.g., K-means, SVM, OC-SVM, was employed to classify the vehicles into three classes: small, midsize, and large. The use of these algorithms in the classification stage allows considering all variations in the geometric vehicle features observed in the training data.

### 5.4. SVM and OC-SVM 

SVM and OC-SVM were the classifiers with the best performance; OC-SVM achieved a global *recall* and an *F-measure* of up to 98.525%, and a *F-measure* of 99.211% for medium size vehicles of video V6. The authors consider that the performance differences between SVM and OC-SVM are due to the parameters selected. In this work, the values of parameter C and η used to evaluate the SVM classifier are {1, 5, 36} and {0.5, 0.65, 0.95}, respectively. The parameter values for evaluating OC-SVM, i.e., η and υ, are {1, 10.5, 15} and {0.001, 0.01, 0.1}, respectively. The misclassification cases were due to unsolved occlusions in the detection stage, particularly in those cases where the vehicles move bumper-to-bumper. In future work, we will consider improving detection with a more efficient occlusion algorithm and other methods for background formation.

Behaviors with variations in the perspective views can be observed in video V2 and V3, where although the camera position changed 20 ft, only the models generated from video V2 were used for the classification stage of both videos, indicating that for certain lateral displacement of the camera, the algorithm is robust. In the K-means algorithm, the value of K = 3. Due to the short length of the training data for small vehicles, the K-means centroids may be biased; thus, the mean of each geometric feature was computed previously, and this information was passed as input to the K-means algorithm. 

### 5.5. 3-D Geometric Feature Space

With the use of Area, Width, and Width/Height ratio of the bounding box—the classification performance was improved with respect to that using only one feature: the area (see Table 6). The geometric features are extracted directly of detected blobs; therefore, the computational cost is lower than those achieved with other features proposed in the state-of-the-art, like grey-level co-ocurrence matrix, texture coarseness, or Histogram of Oriented Gradients.

### 5.6. Real Time Processing 

The average time to process one image frame in our system is less than 30 ms, which proves that our approach can run in real time for videos at 25 fps, and with an average-traffic load of 1.32 vehicles per second and peaks of 4 vehicles per second. In general, the higher the traffic load—particularly with large size vehicles—the higher the measured congestion is the vehicle occlusion index.

In this paper, a high-performance computer vision system is proposed for vehicle detection, tracking, and OC-SVM classification, which has the following advantages:

1. For the GMM based detection stage, the system does not require sample training and camera calibration.
2. Except for ROI, lane-dividing lines, the detection line, and the classification line, it requires no other initialization.
3. A proposed simple algorithm reduces occlusions, particularly in those cases where vehicles move side by side.
4. The use of OC-SVM and a 3D geometric feature space for the classification stage.

## 6. Conclusions

A very high-performance vision system with a single static camera, suitable for an IoT Smart City, for front- and rear-view moving vehicle detection, tracking, counting, and classification was achieved, implemented, and tested. The number and quality of employed metrics outperforms those used in most comparable papers. 

The vehicle occlusion index defined here is a measure of how frequent the occlusion is, and how well the occlusion-handling algorithm performs its function. Our results support that the lower the VOI-Index, the better the performance of the algorithms for detection and classification. 

Experimental results showed that our system performs well in real time with an average traffic flow of 1.32 vehicles per second and traffic load peaks from 2 to 4 vehicles/s on a three-lane road. A mean processing time of about 75% between two consecutive frames was achieved. The best classifiers were with SVM, where OC-SVM with a RBF Kernel successfully classified the vehicles with a high performance, e.g., *recall*, *precision*, and *F-measure* of up to 98.190%; and up to 99.051% for the midsize class. 

The high performance of this system is due to the use of a 3D geometric feature space with side-occlusion handling as an output space of the detection stage (input feature space for the classification), the use of OC-SVM with a RBF Kernel in the classification stage, and the classification is performed in a specific line of the ROI to reduce intra-class differences of the input space.

Finally, an extensive test environment is available for researchers. It has eight videos with 4111 manually labelled ground truth vehicles and a duration of more than 61 min, three places in different countries and under different weather conditions, a mean traffic load of up to 1.32 vehicles/s with traffic load peaks from 2 to 4 vehicles/s (see Figure 8), and a vehicle occlusion index of up to 0.312. 

Open Issues remaining after this study include:

- Develop algorithms for the formation of background with different color spaces and updating is crucial for the different stages of traffic surveillance.
- Develop algorithms for automatic detection of the ROI and the lane-dividing lines.
- Improve algorithms for occlusion caused by high traffic loads, particularly for large vehicles, to increase the detection rate and, consequently, decrease variance of the values of points belonging to the input space for tracking and classification, and to characterize the occlusion by metrics.
- Due to the number of features associated with this problem and the variance of intra-class and interclass feature values, the determination of the optimal number of classes for classification remains an open issue.

## Figures and Tables

**Table 1:** Related works in the detection of vehicles.

Reference	*GT*	Frames	Scenarios	Traffic Load	*DR or Recall*	*Precision*	*F-Measure*
Saunier, N.; Sayed, T. [38] (2006)	302	8360	3	-	88.4	-	-
Hsieh, J.-W.; Yu, S.-H.; Chen, Y.-S.; Hu, W.-F. [20] (2006)	20,443	16,400	3	-	82.16	-	-
Hu, Z.; Wang, C.; Uchimura, K. [35] (2007)	1074	Not indicated	-	-	99.3	-	-
Zhang, W.; Wu, Q. M. J.; Yang, X.; Fang, X. [36] (2008)	427	Not indicated	-	-	93.87–84.43, 100–83.8	-	-
Fang, W.; Zhao, Y.; Yuan, Y.; Liu, K [37] (2011)	226	3500	2	-	86.8, 100	-	-
Arróspide, J.; Salgado, L.; Nieto, M. [35] (2012)	4000	NA	-	-	96.14, 89.92, 94.14	-	-
Pham, H.V.; Lee, B.-R. [21] (2015)	672	18,000	1	-	97.17	-	-
Shirazi, M.S.; Morris, B. [39] (2015)	Not indicated	1080 at 8 fps	3	-	94	-	-
Our System (2017)	4111	92,160 at 25 fps	5	1.34	82.42–99.24	68.7–99.5	74.6–98.3

**Table 2:** Related works in the classification of vehicles.

Reference	Sensors	Scenarios	Input Space	Result	Reported Metrics
Hsieh, J.-W.; Yu, S.-H.; Chen, Y.-S.; Hu, W.-F. [20] (2006)	Camera only	Static side-road camera	Size and the “linearity” of a vehicle	Global TPR of up to 94.8% for cars, minivans, trucks, and van-trucks	*TPR*
Feng, Z.; Mingzhe, W. [9] (2009)	Anisotropic magnetoresistive (AMR) sensor	Vehicle passes through the sensor	Features of wave length, mean, variance, peak, valley, and acreage	86%, 80%, 81%, and 89% *TPR* for big truck, bus, van, and car	*TPR*
Changjun, Z.; Yuzong, C. [10] (2009)	Acoustic signals	Vehicles on the road ramp	Set of frequency feature vectors	95.12% *accuracy* for car, bus, truck, and container truck	*Accuracy*
Chen, Z.; Pears, N.; Freeman, M.; Austin, J. [48] (2014)	Stationary roadside (CCTV) camera	Static side-road camera	Size and width of the blob	88.35%, 69.07%, and 73.47% *TPR* for car, van, and heavy goods vehicles	*TPR*, *TNR*, *FPR*
Moussa, G.S. [46] (2014)	Laser sensor	Top-down laser over road (different scenarios from those presented here.)	Geometric-based features	99.5%, 93.0%, and 97.5% *TPR* for small, midsize, and large	*TPR*
Liang, M.; Huang, X.; Chen, C.H.; Chen, X.; Tokuta, A. [45] (2015)	Camera only	Static side-road camera	Low level features	79.9%, 63.4%, and 92.7%, *TPR* for small, midsize, and large	*TPR*
Lamas-Seco, J.; Castro, P.; Dapena, A.; Vazquez-Araujo, F. [8] (2015)	Inductive Loop detectors	Vehicle passes through the sensor	Fourier Transform of inductive signatures	Global *TPR* of up to 95.82% for small, midsize, and large	*TPR*
Kamkar, S.; Safabakhsh, R. [44] (2016)	Camera only	Static side-road camera	Vehicle length and Grey-Level Co-occurrence matrix features	71.9% Global *TPR* for small, midsize, and large	*TPR*
Our System (2017)	Camera only	Static side-road camera	3-D geometric-based features	Global *TPR* of up to 98.190% for small, midsize, and large	*Recall* or *TPR*, *F-measure*, *Precision*, and VOI-Index

**Table 3:** Videos analyzed in this work.

Video	Frames	Vehicles per Second	Occlusion Index	Recording Place	Vehicle Direction	Weather
V1	16,925	1.24	0.312	Ringroad, Guadalajara, Mexico	Front	Sunny
V2	5400	1.05	0.189	Ringroad, Guadalajara, Mexico	Front	Sunny
V3	3875	0.75	0.124	Ringroad, Guadalajara, Mexico	Front	0 to 20 s Sunny, 21 to 140 s Cloudy
V4	7520	0.88	0.000	M-30, Madrid, Spain	Rear	Sunny
V5	9390	0.63	0.000	M-30, Madrid, Spain	Rear	Cloudy
V6	15,050	1.32	0.249	M6 motorway, England	Front	Cloudy
V7	14,875	1.21	0.203	M6 motorway, England	Front	Cloudy
V8	19,125	1.18	0.202	M6 motorway, England	Front	Cloudy

**Table 4:** Experimental results of the detection stage with occlusion handling.

Video	*GT*	*TP*	*FP*	*FN*	*Detection Rate*	*Precision*	*F-Measure*
V1	842	694	324	148	82.422	68.172	74.623
V2	228	202	104	26	88.596	66.013	75.655
V3	116	103	30	13	88.793	77.44	82.730
V4	264	262	7	2	99.242	97.397	98.311
V5	236	228	1	8	96.610	99.563	98.064
V6	797	761	53	36	95.483	93.488	94.475
V7	725	686	43	39	94.620	94.101	94.360
V8	903	862	82	41	95.459	91.313	93.340

**Table 5:** Experimental results of the classification stage.

Video	Class	Input Space	*TP*	*FP*	*FN*	*Recall*	*Precision*	*F-Measure*
V1	S	179	179	132	0	100.000	57.556	73.061
	M	789	669	20	120	84.790	97.097	90.527
	L	50	16	2	34	32.000	88.888	47.058
	T	1018	864	154	154	84.872	84.872	84.872
V2	S	35	34	26	1	97.142	56.666	71.578
	M	210	177	5	33	84.285	97.252	90.306
	L	61	55	9	6	90.163	85.937	88.000
	T	306	266	40	40	86.928	86.928	86.928
V3	S	11	10	1	1	90.909	90.909	90.909-
	M	97	95	8	2	97.938	92.233	95.000
	L	25	18	1	7	72.000	94.736	81.818
	T	133	123	10	10	92.481	92.481	92.481
V4	S	16	15	12	1	93.750	55.555	69.767
	M	233	222	4	11	95.279	98.230	96.732
	L	20	14	2	6	70.000	87.500	77.777
	T	269	251	18	18	93.308	93.308	93.308
V5	S	3	3	6	0	100.00	33.333	50.000
	M	220	211	0	9	95.909	100.000	97.911
	L	6	4	5	2	66.666	44.444	53.333
	T	229	218	11	11	95.196	95.196	95.196
V6	S	3	2	2	1	66.667	50.000	57.142
	M	766	755	1	11	98.564	99.867	99.211
	L	45	45	9	0	100.000	83.333	90.909
	T	814	802	12	12	98.525	98.525	98.525
V7	S	2	1	3	1	50.000	25.000	33.333
	M	688	676	2	12	98.255	99.705	98.975
	L	39	37	10	2	94.871	78.723	86.046
	T	729	714	15	15	97.942	97.942	97.942
V8	S	5	4	9	1	80.000	30.769	44.444
	M	882	867	3	15	98.299	99.655	98.972
	L	57	55	6	2	96.491	90.163	93.220
	T	944	926	18	18	98.093	98.093	98.093

**Table 6:** Experimental results of the classification stage of videos V6, V7, and V8 using different input spaces and classifiers.

	**Classification with the Thresholds and 1D Feature Input Space**							
Test	Class	Input Space	*TP*	*FP*	*FN*	*Recall*	*Precision*	*F-Measure*
With occlusion handling	S	10	9	474	1	90.000	1.863	3.651
	M	2336	1875	63	461	80.265	96.749	87.739
	L	141	39	27	102	27.659	59.090	37.681
	Total	2487	1923	564	564	77.322	77.322	77.322
	**Classification with K-Means and 3D Feature Input Space**							
Test	Class	Input Space	*TP*	*FP*	*FN*	*Recall*	*Precision*	*F-Measure*
With occlusion handling	S	10	10	247	0	100.00	3.891	7.490
	M	2336	2079	23	257	88.998	98.905	93.690
	L	141	117	11	24	82.978	91.406	86.988
	Total	2487	2206	281	281	88.701	88.701	88.701
	**Classification with SVM and 3D Feature Input Space**							
Test	Class	Input Space	*TP*	*FP*	*FN*	*Recall*	*Precision*	*F-Measure*
With occlusion handling	S	16	16	100	0	100.000	13.793	24.242
	M	2333	2214	4	119	94.899	99.819	97.736
	L	138	133	20	5	96.376	86.928	91.408
	Total	2487	2363	124	124	95.014	95.014	95.014
	**Classification with OC-SVM and 3D Feature Input Space**							
Test	Class	Input Space	*TP*	*FP*	*FN*	*Recall*	*Precision*	*F-Measure*
With occlusion handling	S	10	7	14	3	70.000	33.333	45.161
	M	2336	2298	6	38	98.373	99.739	99.051
	L	141	137	25	4	97.163	84.567	90.429
	Total	2487	2442	45	45	98.190	98.190	98.190

## Appendix A

Links to the video processing files uploaded at YouTube.

V1 https://youtu.be/va0M-2-bobA

V2 https://youtu.be/SeEhrogzXec

V3 https://youtu.be/BDGyB7XDV_E

V4 https://youtu.be/L8rEPmMO4x4

V5 https://youtu.be/csdjk7hhtcE

V6 https://youtu.be/FMLTJGlOs1w

V7 https://youtu.be/CBJ30IUo2x0

V8 https://youtu.be/XGzb8VbpG2E

## Appendix B

Table A1 shows the comparison between the results using an occlusion algorithm in the detection stage or not for videos V6, V7, and V8.

**Table A1:** Experimental results of the detection stage of videos V6, V7, and V8.

Test	Video	*GT*	*TP*	*FP*	*FN*	*Detection Rate*	*Precision*	*F-Measure*
Without occlusion handling	V6	797	653	6	144	81.932	99.089	89.697
	V7	725	624	12	101	86.069	98.113	91.697
	V8	903	755	16	148	83.610	97.924	90.203
	Total	2425	2032	34	393	83.793	98.354	90.492
With occlusion handling	V6	797	761	53	36	95.483	93.488	94.475
	V7	725	686	43	39	94.620	94.101	94.360
	V8	903	862	82	41	95.459	91.313	93.340
	Total	2425	2309	178	116	95.216	92.842	94.014

Table A2 shows the confusion matrix obtained in the classification stage of videos V6, V7, and V8; (a–d) are the confusion matrix of the threshold, K-means, SVM, and OC-SVM methods, respectively.

**Table A2:** Matrix confusion of the classification stage of videos V6, V7, and V8.

Threshold					K-Means				
	S	M	L	T		S	M	L	T
S	9	1	0	10	S	10	0	0	10
M	434	1875	27	2336	M	246	2079	11	2336
L	40	62	39	141	L	1	23	117	141
T				2487	T				2487
(a)					(b)				
SVM					OC-SVM				
	S	M	L	T		S	M	L	T
S	16	0	0	16	S	7	3	0	10
M	99	2214	20	2333	M	13	2298	25	2336
L	1	4	133	138	L	1	3	137	141
T				2487	T				2487
(c)					(d)				
